# Cultivation and characterization of primordial germ cells from blue layer hybrids (Araucana crossbreeds) and generation of germline chimeric chickens

**DOI:** 10.1038/s41598-021-91490-y

**Published:** 2021-06-21

**Authors:** Stefanie Altgilbers, Sabine Klein, Claudia Dierks, Steffen Weigend, Wilfried A. Kues

**Affiliations:** 1 Department of Biotechnology, Institute of Farm Animal Genetics, Friedrich-Loeffler-Institut, 31535 Neustadt, Germany; 2 Department of Genetic Ressources, Institute of Farm Animal Genetics, Friedrich-Loeffler-Institut, 31535 Neustadt, Germany

**Keywords:** Biotechnology, Developmental biology

## Abstract

The chicken (*Gallus gallus*) is one of the most common and widespread domestic species, with an estimated total population of 25 billion birds worldwide. The vast majority of chickens in agriculture originate from hybrid breeding programs and is concentrated on few commercially used high performance lines, whereas numerous local and indigenous breeds are at risk to become extinct. To preserve the genomic resources of rare and endangered chicken breeds innovative methods are necessary. Here, we established a solid workflow for the derivation and biobanking of chicken primordial germ cells (PGCs) from blue layer hybrids. To achieve this, embryos of a cross of heterozygous blue egg layers were sampled to obtain blood derived and gonadal male as well as female PGCs of different genotypes (homozygous, heterozygous and nullizygous blue-allele bearing). The total efficiency of established PGC lines was 45% (47/104) within an average of 49 days until they reached sufficient numbers of cells for cryopreservation. The stem-cell character of the cultivated PGCs was confirmed by SSEA-1 immunostaining, and RT-PCR amplification of the pluripotency- and PGC-specific genes *cPOUV*, *cNANOG*, *cDAZL* and *CVH*. The Sleeping Beauty transposon system allowed to generate a stable integration of a Venus fluorophore reporter into the chicken genome. Finally, we demonstrated that, after re-transfer into chicken embryos, Venus-positive PGCs migrated and colonized the forming gonads. Semen samples of 13 raised cell chimeric roosters were analyzed by flow cytometry for the efficiency of germline colonization by the transferred PGCs carrying the Venus reporter and their proper differentiation into vital spermatids. Thus, we provide a proof-of-concept study for the potential use of PGCs for the cryobanking of rare breeds or rare alleles.

## Introduction

Chicken primordial germ cells (PGCs) are the unipotent precursors of sperm cells and ova. In all vertebrates PGCs have an extragonadal origin, but show different segregation- and migration patterns among the classes^1^. In chicken embryos, PGCs shift from their epiblast origin during the primitive streak progression into an extraembryonic region referred to as germinal crescent, which is the area anterior to the head fold^2^. With reference to the Hamburger and Hamilton (HH) staging of normal chicken development, PGCs migrate from the germinal crescent along the forming extra-embryonic into the intra-embryonic blood vessels at HH 11–12^2, 3^. For a short time frame, they circulate in the embryonic blood (HH13–HH17) and finally settle down into the genital ridges (HH28–HH30)^3, 4^. During these stages of embryonic development, the PGCs can be isolated from the blood or the gonads and their number can be enriched in vitro under defined cell culture conditions. Currently, a long-term culture without a feeder layer is only established for avian PGCs^5–7^. This recently developed culture system allows maintaining of the germline character, in vitro propagation and re-transplantation of PGCs into host embryos^7, 8^.

Due to the commercial focus on a few high-performance lines and the predominant use of commercial hybrids in poultry production worldwide, there is a risk that genetic diversity in chickens will be lost. A limited number of founder breeds were involved for white and brown egg layer lines, mainly the White Leghorn, Rhode Island Red and White Plymouth Rock breeds^9, 10^. Even though fancy breeds are maintained by hobby breeders in some countries, the genetic diversity of local and indigenous breeds is threatened worldwide^10, 11^. The cryopreservation of rooster semen is an established method, but cannot preserve specific female genetic elements, i. e. the W-sex chromosome and the mitochondrial genomes^12, 13^ since the males are the homogametic (ZZ) sex in chicken^14^. The chicken mitochondrial genome is a circular molecule compromising approximately 16.8 kilobases (kb), which encodes 13 proteins, two rRNAs, and 22 tRNAs^15^. However mitochondria harbor unique biochemical pathways for the generation of chemical energy, the synthesis of important hormones^16^, and ammonia clearance^17^. Even single base pair changes in mitochondrial genes may have massive effects on production traits^18^. The diploid PGCs are replication competent and allow for extensive cell expansion, thus potentially allowing the preservation of the whole genome from minute quantities of starting material. The reconstitution of a rare breed or rare alleles with the help of PGCs becomes attractive particularly now, when genetically sterile chicken can act as surrogate hosts for PGC transplantation^13, 19^. Through transmitting the genetic information, the potential of PGCs for avian gene preservation is tremendous.

In this study, we choose to cultivate PGCs from hybrids between Araucana and a White Leghorn chicken line to establish a workflow for the cryobanking of chicken male and female genotypes carrying a rare allele. The phenotype of the blue-green eggshell color of the Araucana breed is due to the incorporation of biliverdin during eggshell formation in the shell gland of the oviduct^20^. A provirus (EAV-HP) insertion in the 5' flanking region of the SLCO1B3 gene in Araucana chicken is supposed to be causative for the blue eggshell color in this breed^21, 22^. The SLCO1B3 gene is overexpressed in the eggshell gland and oviduct and codes for a solute carrier molecule, which transports bile salts such as biliverdin^21, 22^. High amounts of biliverdin in the shell gland during the calcification process lead to a blue eggshell color on the inside and the outside.

Here we describe a workflow to gain, cultivate, and cryopreserve PGCs bearing the rare allele of blue eggshell color and to test these cell lineages for their ability to colonize the gonads of non-sterile host embryos. The long-term suspension culture of chicken PGCs without a feeder layer facilitates the still challenging cultivation of these unipotent and diploid stem cells^7^. To confirm the germline competency and to track the migratory competence of PGCs after long-term expansion in vitro, we stably integrated a Venus reporter with the Sleeping Beauty (SB) transposon system^23^. The SB-system originates in fish (salmonids) and belongs to the Tc1/mariner superfamily^24^. This engineered binary transposon system was successfully used previously in mice^23^, pigs^25^, rabbits^26^ and cows^27^.

## Results

### Derivation and culture behavior of PGCs

Embryonated eggs for the PGC derivation were generated from chickens of an intercross generation. This intercross was established at the end of a backcrossing experiment to demonstrate the marker-assisted breeding transfer of a specific trait, the blue eggshell color, into a high performing white egg layer chicken line^28^. In brief, first a F1 generation was generated by mating homozygous blue-allele bearing rooster with White Leghorn hens, followed by two generation of backcrossing to the White Leghorn line. Heterozygous blue-allele bearing chickens from the second backcross generation were used for the production of fertilized eggs (Intercross breeding).

Embryonated eggs were used to isolate PGCs during the migratory phase of Hamburger and Hamilton stages (HH) 14–16, and shortly after colonization of the forming gonads (HH 28–29)^3^ to compare the efficiencies of cell line establishment of the different origins.

**Figure 1:**
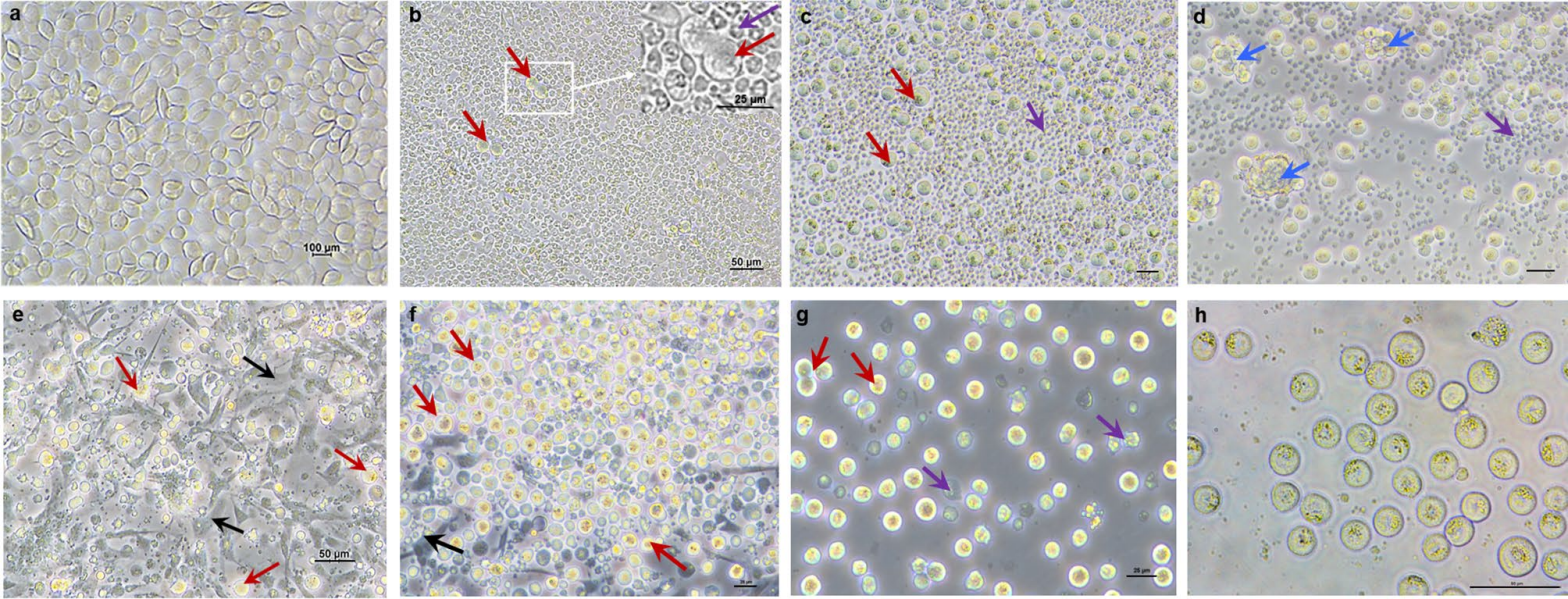
Establishment of PGC lines from embryonic blood and gonads. (**a**) At day 1 of in vitro culture of embryonic blood. Haemopoietic precursor cells dominate the culture (scale bar: 10 µm). (**b**) At day 4 of in vitro culture of embryonic blood, two dividing PGCs (Doublets) are visible (scale bar: 50 µm). (**c**) At day 9 of in vitro culture of blood-derived PGCs which now dominate the cell culture (scale bar: 25 µm). (**d**) At day 13 of in vitro culture of a female blood-derived PGC-lineage which tends to form cluster (scale bar: 25 µm). (**e**) At day 1 of in vitro culture of gonadal-derived PGCs. PGCs are loosely attached to the surface of the gonadal stroma cells (scale bar: 50 µm). (**f**) At day 3 of in vitro culture of gonadal-derived and already proliferated PGCs (scale bar: 25 µm). (**g**) At day 10 of in vitro culture of gonadal-derived PGCs. Subculturing eliminates the adherent stroma cells but some cell debris is still present (scale bar: 25 µm). (**h**) Established male PGC-lineage with lipid vacuoles in the cytoplasm (as described here:^8^) (scale bar: 50 µm). red arrows: PGC, black arrows: gonadal stroma cell, blue arrows: cell cluster, violet arrows: cell debris.

In the first days of in vitro culture, the blood-derived samples were dominated by haemopoietic progenitor cells from the embryonic blood (Fig. 1a). Hence, no PGCs were visible during the first days. From day four on the PGCs started to proliferate, while the haemopoietic progenitor cells began to disappear due to non-appropriate culture conditions (Fig. 1b–d). As a typical growth sign for PGC proliferation the appearance of round cell doublets was detected (Fig. 1b).

In gonadal samples, the culture background was dominated by adherent gonadal stroma cells. The immediately visible PGCs were loosely attached to these cells (Fig. 1e,f). While transferring the medium including the PGCs into a new well of a 48-well cell culture plate most of the attached cells remained in the previous well. Soon, the cell culture became free of adherent cells and only a few dead cells were still detectable (Fig. 1g,h).

Interestingly, some cell lineages, especially the female ones, tended to form cell cluster and the cells of these cluster often atrophied (Fig. 1d). These small aggregates appeared throughout the cultivation process and also remained after successful establishment. A cell line derived from a single embryo was defined as an established cell lineage when it reached 1.5 × 10^6^ cells in total.

Starting with 104 eggs, we were able to establish a total of 47 cell lines consisting of 16 female and 31 male PGCs. Sexing and genotyping were done after establishment of the cell line. Genotyping resulted in 21 heterozygous-blue, 17 homozygous-blue and 9 nullizygous-blue allele bearing cell lines (Table 1). A higher proportion of cell lines was established from gonadal PGCs (60%) than from blood-derived PGCs (34%). However, comparable proportions of sex and blue egg allele carrying cell lines were obtained from both origins. On average, it took 49 days to establish a blood-derived or gonadal cell line. The fastest growing cell lines required 35 days and the slowest growing ones 66 days to reach 1.5 × 10^6^ cells (Table S1). The slowest growers were female blood-derived PGCs. All 47 PGC cell lines were cryopreserved. For this purpose, 1–2 aliquots with 1.5–2 × 10^6^ of each cell line were frozen to − 80 °C in a *CoolCell* freezing container and transferred to liquid nitrogen the next day for long-term storage. Afterwards we randomly choose and thawed six cell lines, one male and one female for each genotype we established, to illustrate some growth curves and determine doubling time. We set the cell count to 100.000 and followed the growth over four days. Doubling times between 25 and 36 h were found (Table S2, Fig. S1). In our case, the male and also the female homozygous blue-allele bearing cell line needed less time to double than the other genotypes. The three female counted cell lines had comparable doubling times to their male counterparts.

**Table 1:** Derivation and genotyping of PGCs from different sources.

Source	No. of sample	No. of established cell lines (% efficiency)	Sex (% of efficiency)		Genotype (% of efficiency)		
			Female	Male	Heterozygous blue-allele	Homozygous blue-allele	Nullizygous blue-allele
Gonads	43	26 (60)	8 (31)	18 (69)	11 (42)	8 (31)	7 (27)
Blood	61	21 (34)	8 (38)	13 (62)	10 (48)	9 (43)	2 (9)
Total	104	47 (45)	16 (34)	31 (66)	21 (45)	17 (36)	9 (19)

### Characterization of PGCs

**Figure 2:**
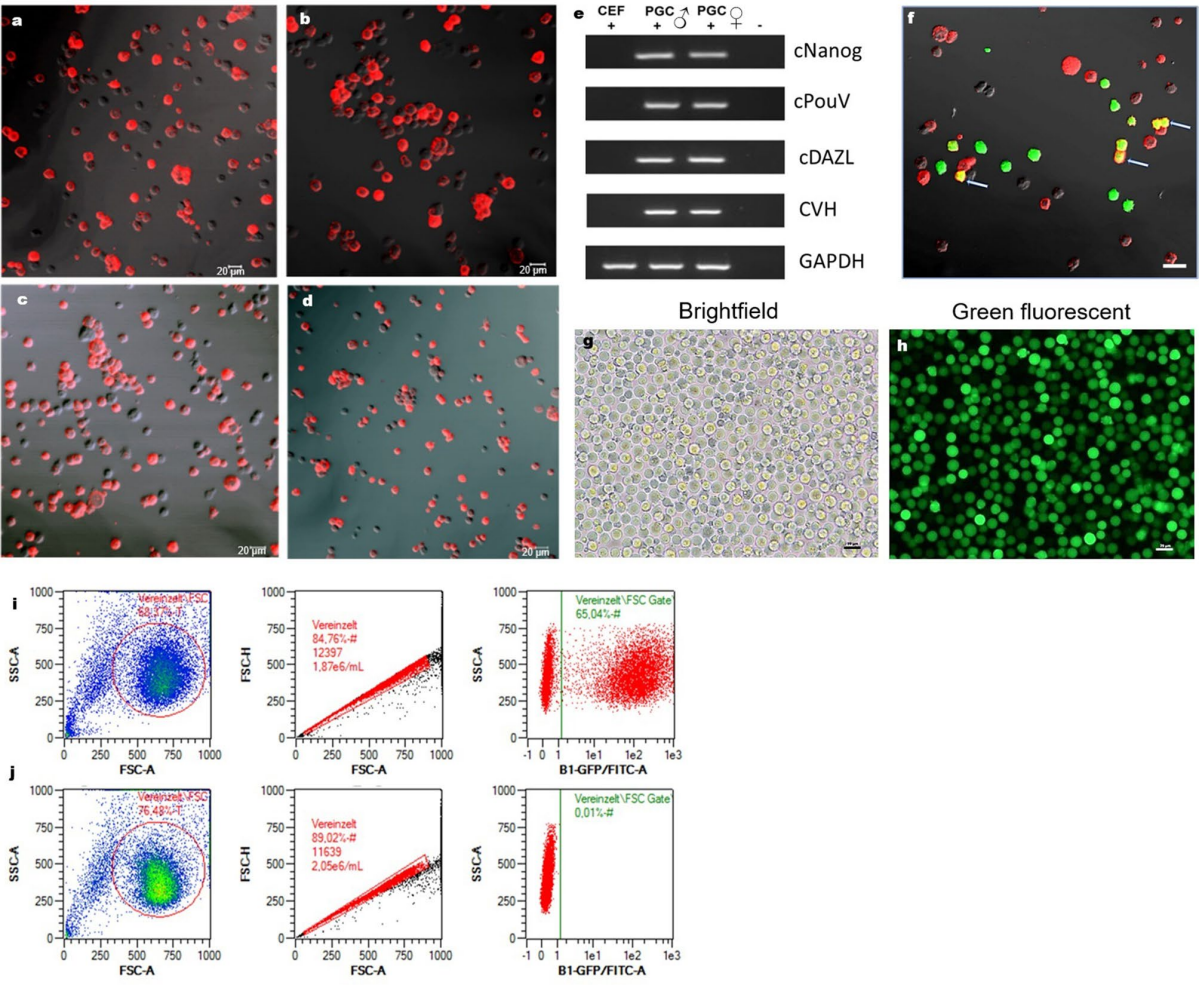
Characterization of Venus-positive PGCs. (**a**–**d**) Immunofluorescence of SSEA-1 on the surface of PGCs (scale bar = 20 µm). (**a**) male PGC cell line (homozygous blue-allele). (**b**) female PGC cell line (homozygous blue-allele). (**c**) male PGC cell line (heterozygous blue-allele). (**d**) male PGC cell line (nullizygous blue-allele). (**e**) Expression of marker genes in male and female PGCs, CEF: chicken embryo fibroblasts, pluripotency marker: *cNANOG*, *cPOUV*, stem cell marker: *cDAZL*, *CVH*, housekeeping gene: *GAPDH*, blank—no cDNA control. (**f**) Immunofluorescence of SSEA-1 (arrows: co-expression of SSEA-1 and Venus-protein), (scale bar: 20 µm). (**g** + **h**) Expression of the Venus-gene in cultured chicken PGCs, (**g**) Brightfield, (**h**) Venus^+^ PGCs (scale bar: 25 µm). (**i** + **j**) Venus-fluorescence of PGCs transfected with Venus-transposon in presence and absence of transposase; (**i**) post-transfection status of PGCs transfected with Venus-transposon and SB-transposase. 65% of PGCs with transient integrated Venus-gene; (**j**) post-transfection status of PGCs transfected with Venus-transposon and without SB-transposase. No Venus-fluorescence is detectable.

The established gonadal and blood-derived PGCs were all round-shaped and approximately 15–20 µm in size. Refractive lipid vacuoles in the cytoplasm were clearly evident (Fig. 1h). To confirm the stem cell character of the in vitro cultivated PGCs, SSEA-1 immunostaining was done in one cell line from each genotype (Fig. 2a–d). The SSEA-1 epitope was expressed on the surface of the stained PGCs. Additionally, we examined the expression of the PGC-specific genes *cPOUV*, *cNANOG*, *cDAZL* and *CVH* in PGCs and in chicken embryo fibroblasts (Fig. 2e). The pluripotency markers *cPOUV* and *cNANOG* as well as the specific PGC stem cell markers *cDAZL* and *CVH* were only expressed in PGCs. No expression was found in chicken embryo fibroblasts (CEFs), with the exception of the housekeeping gene *GAPDH*.

### Stable transfection of PGCs with the Sleeping Beauty transposon-system

To enable direct tracking of the PGCs for potential re-transplantations into host embryos, the cells were transfected with a ubiquitously expressed Venus-SB-transposon plasmid either with or without the Sleeping Beauty transposase helper plasmid and cultivated for two weeks to provide a proof of stable integration. Co-expression of Venus-protein and SSEA-1, a marker expressed in stem cells, was present in post-transfected PGCs (Fig. 2f). The initial transfection efficiencies for the different cell lines varied between 65 and 85% and the Venus-transposon was strongly expressed in the cells (Fig. 2g–i). In the absence of transposase, no Venus-positive PGCs were detected by flow cytometry post-transfection (Fig. 2j). After 2 weeks, the efficiency of stable transfection varied between 7 and 12.9% (Fig. S2). For the production of adult germline chimeras, the Venus-positive PGCs were selected by fluorescence activated cell sorting before microinjection into recipient embryos.

### Migration efficiency of Venus-positive PGCs

**Figure 3:**
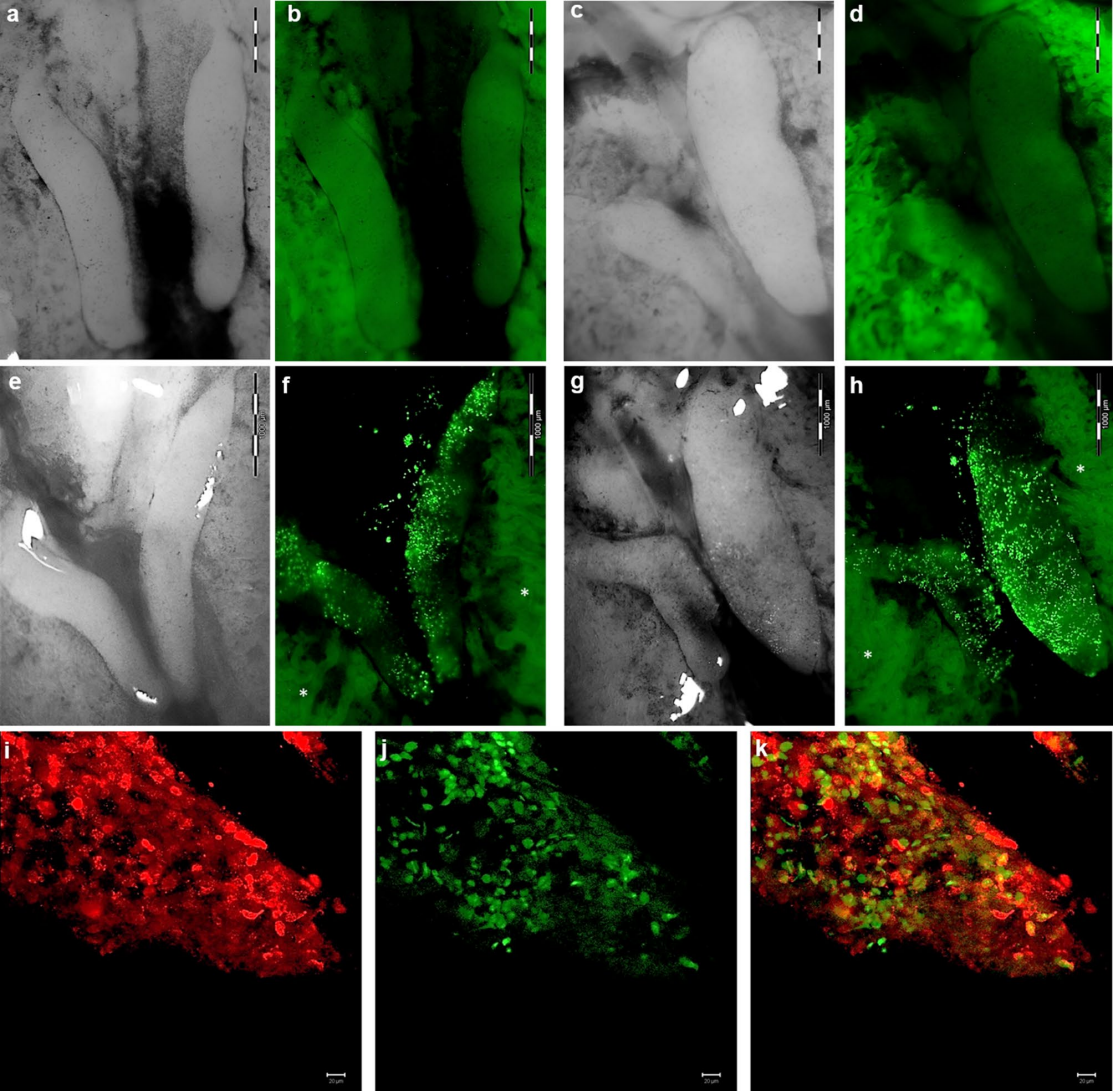
Venus reporter transgenic PGCs in chicken gonads at day 10 of embryonic development. (**a**) Microscopic image of male gonads (Brightfield); (**b**) autofluorescence of male gonads and mesonephros (wildtype, Venus-negative); (**c**) microscopic image of female gonads (Brightfield); (**d**) autofluorescence of male gonads and mesonephros (wildtype, Venus-negative); (**e**) microscopic image of male gonads (Brightfield); (**f**) green fluorescence of male gonads; (**g**) microscopic image of female gonads (ovary and rudimentary right ovary); (**h**) green fluorescence of female gonads; (**i**) immunofluorescence of SSEA-1 in male gonad; (**j**) Venus-fluorescence of PGCs in the colonized male gonad; (**k**) co-expression of SSEA-1 and Venus-protein in PGCs in a male gonad (Merge). *Autofluorescence of the mesonephros, (**a**–**h**: scale bar: 1000 µm; **l**–**k**: scale bar: 20 µm).

We set up a hatching experiment and microinjected the male heterozygous blue-allele bearing and Venus transfected PGCs into White Leghorn recipient embryos at 2.5 days of development (HH14–16). At this stage, migratory primordial germ cells peak in the blood circulation^29^. We did not examine the sex of the recipient embryos before injection since we know from previous studies that male PGCs also colonize the female gonads even though they do not form functional gametes because meiosis will not be completed^5, 8, 30^. The gonads of the recipient embryos, which died during the incubation process, were checked for the colonization with the Venus-positive PGCs. After 21 days of incubation 29 chicks hatched. We injected 76 embryos and found 92% Venus-positive gonads from 63 injected embryos, including 16 hatched female chicks, which were sacrificed in the first week after hatch (Table 2). Thirteen male chicks were raised to sexual maturity. Based on the fluorescence microscopic image Venus-positive PGCs were distributed over the entire male and female gonads (Fig. 3a–h). Some gonads with the highest amount of Venus-positive PGCs were fixed and co-expression of SSEA-1 and Venus-protein was assayed (Fig. 3i–k). The confocal microscopic images showed that the co-expressing donor PGCs were evenly distributed alongside Venus-negative host PGCs in the germ cell niches of the embryonic gonad.

**Table 2:** Migration efficiency of Venus-positive PGCs.

Cell line			No. of Venus-positive gonads	No. of embryos injected	No. of hatched chicks (%)	No. and sex of hatched chicks	
Days in culture	Sex	Genotype				Male	Female
96	Male	Heterozygous (blue-allele)	58 (92%)	76	29 (38)	13	16

Additionally, we microinjected the male homozygous and nullizygous blue-allele bearing PGCs into White Leghorn recipients. We examined all gonads at day 10 of embryonic development for the colonization with the Venus-positive PGCs (Fig. S3). PGCs of both genotypes colonized the gonads of 99–100% of the injected embryos surviving to day 10 (Table S3). Male and female gonads were equally colonized by the male Venus-positive PGCs.

### Generation of germline chimeric rooster and Venus-positive sperm evaluation

**Figure 4:**
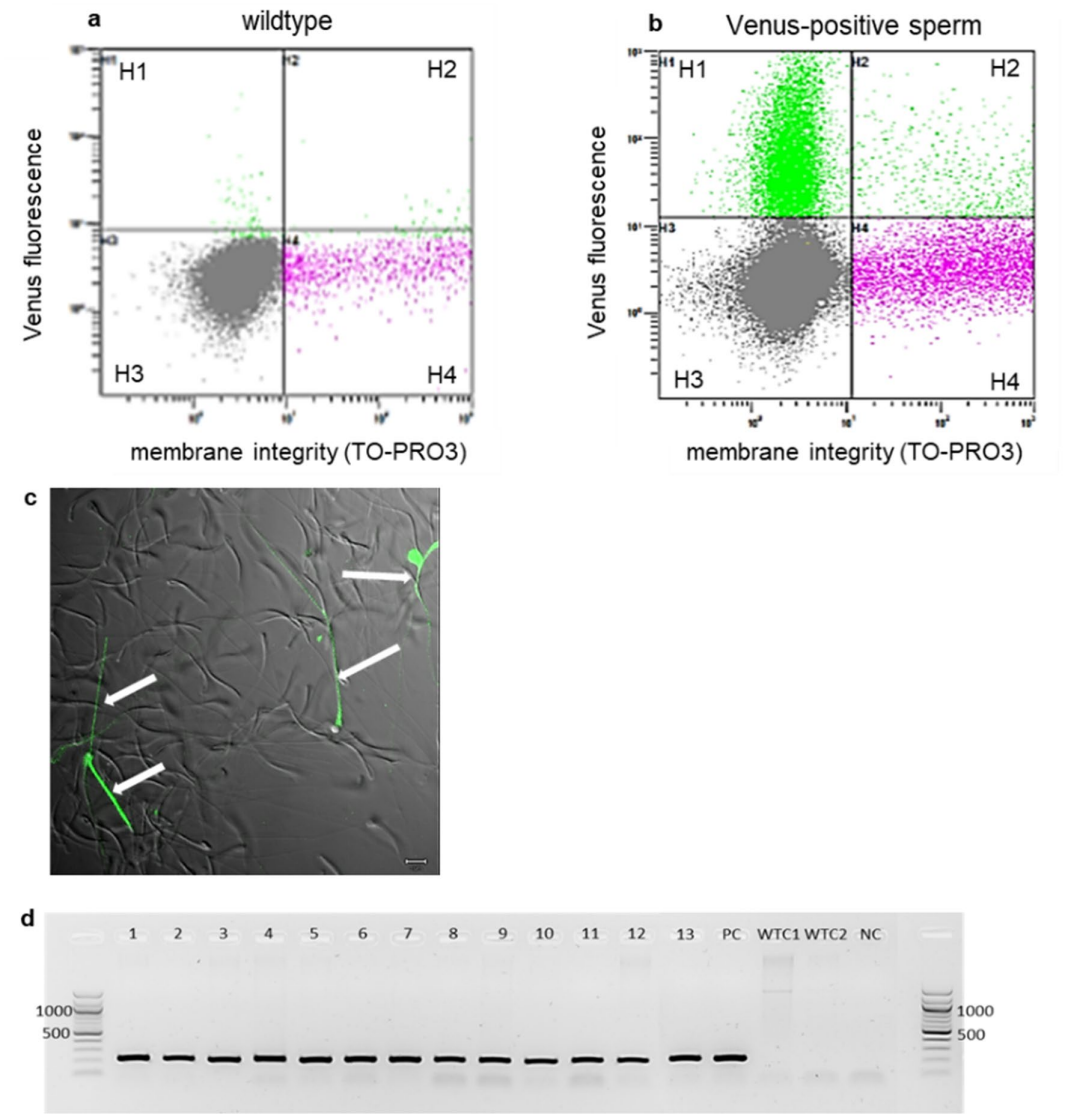
Venus fluorescence of semen from germline-chimeric rooster. (**a** + **b**) Scatterplot image of flow cytometry data from one germline chimeric rooster in comparison to a wildtype control, H1 = Venus-positive and vital, H2 = Venus-positive and non-vital, H3 = Venus-negative and vital, H4 = Venus-negative and non-vital; (**a**) wildtype rooster non-transfected (Venus-negative control); (**b**) Venus transfected germline chimera with the highest amount of vital and Venus-positive sperm amongst the tested rooster (H1); (**c**) Phase contrast and fluorescence of sperms (Merge), Venus-positive sperms (white arrows) (scale bar: 5 µm); (**d**) Venus-PCR of 13 male germline chimeras (1–13), PC = positive control, NC = water sample, WTC = wildtype control (Venus-negative control).

To assess the full developmental capacity of the cryopreserved PGCs, a male heterozygous blue-allele bearing and Venus-positive PGC line was used for the hatching experiment described above. The cell lineage was cultivated in vitro for 96 days. From the 29 healthy chicks, the 13 male chicks were raised over 6 months to reach sexual maturity. Since we used male PGCs for the injection, we raised only the male chicks. These reach sexual maturity earlier than the females and by mating one male with several females more offspring can be obtained^31^. The semen of the germline chimeras was collected, and the presence of Venus-positive sperms was tested by flow cytometry. For this purpose, five ejaculates of each rooster were collected within a weekly interval. The membrane integrity of sperm cells was tested additionally to the Venus reporter presence (Fig. 4b). The results showed that the majority of the Venus-positive sperms had intact membranes (Fig. 4b, H1), while only less Venus-positive sperms were not intact and vital anymore (Fig. 4b, H2). Additionally, the sperm DNA was extracted and a Venus-PCR was performed, which confirmed the presence of the Venus-reporter gene in each of the 13 samples (Fig. 4d). The results showed that all of the 13 roosters produced intact Venus-positive sperms, but at relative low ratios (0.1–7.8%, Table 3). Only the roosters with 2.5% Venus-positive sperms and more were used for germline transmission testing (Table 3, *). Germline transmission is here defined as a process where donor derived PGCs contribute to the gametes of the germline chimeric rooster (recipient) and are passed to its offspring (transgenic chicken). One rooster was not mated to wildtype hens because of low sperm quality and amount (Rooster ID 1961). All the other roosters were fertile and produced healthy wildtype offspring. However, when mated to wildtype hens, the seven roosters with the highest frequency of Venus-positive sperms did not show germline transmission.

**Table 3:** Mean proportion of Venus-positive sperms per rooster (${\overline{\text{x}}}$ ± SD) and number of tested offspring in 14 roosters including one wildtype rooster (non-transfected) as Venus-negative control (NC).

Rooster ID	Venus-positiv sperms %	No. of fertile eggs	No. of Venus-positive embryos
1923*	6.3 ± 1.0	546	0
1933*	5.6 ± 1.7	489	0
1948*	7.0 ± 1.0	567	0
1954	0.7 ± 0.4	–	–
1960*	2.5 ± 1.2	80	0
1961	4.9 ± 1.8	–	–
1974*	5.2 ± 1.1	463	0
1991*	5.3 ± 0.5	502	0
1929	2.4 ± 1.2	–	–
1947	1.7 ± 0.3	–	–
1957*	7.8 ± 1.3	552	0
1966	0.7 ± 0.2	–	–
1969	0.1 ± 0.0	–	–
6479 (NC)	0.1 ± 0.1	–	–

*Rooster tested for germline transmission.

## Discussion

Our results demonstrate that PGCs can be obtained from the embryonic blood or the gonads from a crossbreed of heterozygous blue-allele bearing chickens. The established workflow can be easily adopted to preserve other rare breeds and rare alleles by PGC-mediated biobanking. For chicken, cryobanking of PGCs is an extension to sperm preservation^32^. It would be a benefit to capture rare alleles to be prepared for adapting breeds to future environments or market demands. This proof-of-concept study shows how chicken PGCs bearing a rare allele, can be cultured, cryopreserved, transfected and re-transferred into chicken embryos forming donor-derived sperms in the adult recipients.

First of all, it should be taken into account, that not every embryo gives rise to an established cell line^31^. The efficiency of obtaining PGC cell lines here reached 45%. This is in accordance with previous reports where an efficiency of line establishment of 40–61% was gained^7, 13^. As reported before, male and female PGCs seem to have different growth requirements because the female PGCs often tend to form cell cluster and seem to grow slower than their male counterparts (Fig. 1d; and^31^). Under our conditions, male and female PGCs were successfully cultured in vitro with 66% and 34% efficiency, respectively. Meanwhile, Woodcock et al. improved the derivation rate for female PGCs (61 vs. 30%) using medium supplemented with ovotransferrin^13^. In our study, it took 49 days in average to establish a PGC cell line (1.5 × 10^6^ cells), which is comparable with data from the literature^13, 33^. PGC growth is almost exponential, which indicates that the success of gonadal preparation and tissue separation or the amount of blood that could be taken from the embryonic veins is tremendously important. The higher the initial cell concentration, the faster a cell line can be established^34^. More PGC lines were obtained from gonadal preparation than from blood punctation of the chicken embryos. Since 0.02% of the blood cells and 2% of the gonadal cells are PGCs, the chance to catch proliferating cells is higher for gonadal preparations^35, 36^. Although we were able to establish fewer female cell lines overall, the doubling times are comparable to the male cell lines. That female cells generally grow more slowly could not be observed here. Other groups concluded that the doubling time is genotype dependent^34^. In our case, there is an indication that the homozygous blue-allele bearing PGCs might need less time to double.

We confirmed that the cultured female and male PGCs retained a germ cell identity by performing RT-PCR of the pluripotency- and PGC-specific genes (*cNANOG*, *cPOUV* and *cDAZL, CVH*), which is in agreement with data from previous studies^7, 8^. The stem cell marker SSEA-1, although not solely chicken PGC specific^37^, was stably expressed in all cell lines from each genotype, confirming the stem cell status of the in vitro PGCs.

Using the SB-system we reached high transient transfection efficiencies of 65–85%. Even though the stable transfection rate is much lower (7–12%), our results showed that PGCs do well tolerate electroporation of SB plasmids. The Sleeping Beauty transposon system seems to be a suitable tool to integrate foreign DNA into the chicken genome of PGCs, although the stable transfection efficiency still needs to be improved. PGCs and stem cells in general are described as hard to transfect and edit because double strand brakes are not well tolerated^38, 39^. The stability of the genetic information which is transmitted by the PGCs to the offspring is of tremendous importance, since manipulation of the genome might lead to apoptosis^40^. PGCs, which stably express the Venus-transposon after being cultured for two weeks were positively selected by fluorescence activated cell sorting, and subsequently these Venus-labelled PGCs were successfully used for re-transfer experiments into chicken embryos.

After re-transfer of the stably transfected PGCs, they effectively colonized the gonads of the chicken embryos. We found that the proportion of Venus-positive gonads in all three injected genotypes was about 92–100%. This is consistent with other studies using reporter carrying PGCs^41, 42^. The Venus-transfected PGCs do not lose their ability to migrate from the peripheral blood into the developing gonads of the embryos. Furthermore, the long-term in vitro culture of these PGCs does not cause loss of the germ cell properties. The co-expression of the pluripotency marker SSEA-1 and Venus-protein showed that the foreign donor PGCs can effectively settle down in the early seminiferous tubules of the testes along with the embryo's own PGCs. In general, estimating the migration efficiency of PGCs in chicken gonads is helpful, to select the best gonad-colonizing cell lines for hatching experiments. Even after the injection of male PGCs, the colonization of the embryonic female gonads can be assessed, since the PGCs of the male sex also colonize and divide in the female gonads and vice versa^5, 8, 30^.

After verified gonadal migration, one male heterozygous blue-allele bearing PGC lineage was tested for the ability to undergo full spermatogenesis. At the age of six months each male germline chimera produced vital and intact Venus-positive and Venus-negative sperms (Fig. 4; Table 3). The fertilization was higher than 90% for all roosters but one. However, in our case no donor-derived offspring could be obtained even though we observed high gonadal colonization rates and proper differentiated sperms. In previous studies it was shown that not each cell lineage gives rise to transgenic offspring^5, 8, 43^. The reason for that is still not known. However, it appears that the donor-derived sperms cannot compete with the endogenous sperms. A higher ratio of Venus-positive sperms relative to all sperm cells may improve the germline transmission. As we showed even a good gonadal colonization is no guarantee for germline transmission. Several meiosis checkpoints during spermatogenesis could possibly have induced apoptosis of our Venus-positive cells during this process. Perhaps the donor embryo from which the cell line was derived already had PGCs with mutations that might have reduced spermatogenesis of the Venus-positive sperm. Maybe the cultivation time has impact on mutation rates and development of abnormalities influencing fertility^13, 44^. Unfortunately, the analysis of a karyotype in birds is challenging due to the characteristics of the chicken karyotype with the presence of some so-called macrochromosomes and many microchromosomes, the latter of which are challenging to be distinguished. Especially, micro-deletion or other micro-rearrangements are unlikely to be detected. Alternatively, it has been shown that genome stability of in vitro propagated PGCs can be done by analyzation of de novo SNV formation, but therefore single cells must be established which is also challenging for chicken PGCs^13^. Whole genome sequencing data of more than one single cell clone should then be compared to the somatic DNA from the original embryo to get reliable results.

Until now, there is no way to predict the efficiency of germline transmission of one PGC lineage. The most promising approach to improve the transmission efficiency of donor PGCs are genetically engineered, sterile recipients^13, 19^. Further investigations concerning the transcriptomic differences between male and female or gonadal and blood-derived PGCs should be done, for example by RNA-sequencing. Hints from these data could help to improve the in vitro conditions for PGC cultivation with the aim to gain stable germline competence comparable to in vivo PGCs.

Since in chicken the males are the homogametic sex, it needs a multigenerational breeding program to reconstitute a breed only with the use of sperm^45^. The reconstitution of a breed using PGCs is based on the production of germline chimeras which can be mated with other chimeras, living individuals or artificially inseminated with stored sperm^46^, which will drastically shorten the time to reconstitute a breed or transfer a rare allele into a recipient chicken line.

## Materials and methods

### Animal experiments

Animals were maintained and handled according to the German laws regulating animal welfare, and genetically modified organisms. The experiments were approved by an external ethics committee (Niedersächsisches Landesamt für Verbraucherschutz und Lebensmittelsicherheit, AZ 33.19.42502-04-17/2432). The study is reported in accordance with the ARRIVE guidelines.

Fertilized eggs from the crossbreed of heterozygous blue egg layers were gifted by Lohmann breeders. These eggs derived from a case study which was part of the EU-project (IMAGE)^28^.

Recipient embryos for PGC re-transfer were generated by mating Lohmann LSL chickens (Geflügelzucht Horstman GmbH, Germany).

### PGC derivation and culture conditions

Heterozygous animals carrying the blue egg allele on a White leghorn background line^28^ were mated by artificial insemination and fertile eggs were incubated for 65 h to obtain Hamburger and Hamilton stages (HH) 14–16, or for 6 days to obtain HH 28–29. The incubated embryos, still sitting on the top of the yolk, were transferred into a weighing pan and handled under a stereomicroscope. For obtaining the embryonic blood a glass capillary (Ø 35–40 µm) was fixed in a manual microinjector (Celltram 4r Oil, Eppendorf). From HH 14–16 embryos a minimum of 1 µl of embryonic blood of a single embryo was placed directly into 300 µl of FGF-activin-chicken serum-medium (FAcs-medium) and transferred into one well of a 48-well plate.

The gonads of a single embryo were each separated from the mesonephros, transferred into phosphate buffered saline (PBS) with 0.1% bovine serum albumin (BSA) and the tissue was dissolved in 0.25% Trypsin–EDTA for 15 min as described by Collarini et al. (2019). Afterwards the reaction was stopped with PGC-medium (FAcs-medium) and the cells were pelleted. The cell pellet was resuspended in PGC-medium and transferred into one well of a 24-well plate for 4 h to allow the adherent cells to attach. The supernatant was then transferred into one well of a 48-well plate.

The PGCs were cultured in suspension without a feeder-layer and sub-cultured as described^47^. The cells were passed to the next largest well-plate when becoming dense. Approximately every second day two-thirds of the medium were changed until the PGCs reached sufficient cell numbers for cryopreservation.

The composition of the used FAcs-medium was previously described by Whyte et al. (2015). The customized avian KO-DMEM (CaCl_2_-free, 12.0 mM glucose, 250 mOsm) produced by ThermoFisher scientific was used as the basal medium supplemented with 1× B-27 supplement, 2.0 mM GlutaMax, 1×  NEAA, 0.1 mM ß-mercaptoethanol, 1×  nucleosides, 0.4 mM pyruvate, 0.2% ovalbumin (Sigma), 0.1 mg/ml sodium heparin (Sigma), 0.15 mM calcium chloride, 12.5 ng/ml human activin A (PeproTech), 4 ng/ml basic fibroblast growth factor (PeproTech), and 0.2% chicken serum (ThermoFisher scientific). PGC lines were expanded to 1.5–2 × 10^6^ cells and cryopreserved in liquid nitrogen with a CO_2_-independent medium (ThermoFisher scientific) supplemented with 2.2 mM Glutamax (ThermoFisher scientific), 10% fetal calf serum (FCS), 1% penicillin/streptomycin (10.000 I.U./10.000 µg/ml) and 10% DMSO. The supplemented CO_2_-independent medium without DMSO, referred to as ‘manipulations-medium’, was also used for the injections into the chicken embryos.

### RNA isolation and cDNA synthesis

Total RNA was isolated from pelleted PGCs (1 × 10^6^) using 1 ml TRIzol Reagent (ThermoFisher scientific) and phenol–chloroform extraction as described^48^. For cDNA synthesis the QuantiTect Reverse Transcription-Kit (Qiagen), including oligodT and random primer, was used according to the manufacturer's guidelines.

### Reverse transcription PCR

PCR conditions were 95 °C for 2 min, 94 °C for 45 s, 60 °C for 45 s, 72 °C for 45 s and a final extension of 72 °C for 5 min for 32 cycles (Promega GoTaq Polymerase). Reaction products were resolved using a 1.5% ultrapure agarose (Invitrogen) gel electrophoresis run at 80 V for 45 min in 1× TBE-buffer, and visualized using a transilluminator. Intron-spanning primer are listed in Table S4.

### Immunohistochemistry on PGCs and embryonic chicken gonads

PGCs were pelleted and were fixed on a microscope slide. The gonads (maximum day-10 gonads) were fixed using 4% formaldehyde in 0.2× PBS, and mounted on a microscopic slide. The embryonic day-10 gonads fixed equally, but treated as whole mounts in suspension. PGCs and gonadal tissue were permeabilized with 0.4% Triton X-100 in 0.2×  PBS and washed with 0.2×  PBS. 10% Normal Goat Serum in PBS was used as blocking solution for 30 min or 2 h for PGCs and gonads, respectively. Samples were incubated with the primary antibody mouse anti-SSEA-1 (1:128, Developmental Studies Hybridoma Bank) for 20 h, and for 7 d with twice antibody changes, at 4 °C for PGCs and gonads, respectively. Samples were washed and incubated with Cy5-conjugated goat anti-mouse IgM secondary antibody (1:600, Merck) for 90 min and 3 d, respectively. The fluorescence was imaged at a confocal microscope (LSM 510, Carl ZEISS Microimaging GmbH). Images were captured using AIM software.

### Transfection of PGCs

1.5 × 10^6^ PGCs were suspended in 200 µl Opti-MEM with 10 µg plasmid-DNA. The Sleeping Beauty Transposase (pCMV-SB100X) and Venus-Plasmid (pT2/VenusRMCE) were co-electroporated (1:6 molar ratio)^23^. Eight square wave pulses (350 V, 100 µsec, interval 200 ms) were given (ECM 830 Square Wave Electroporation System, BTX) as described^5^. Alternatively, the Neon Transfection System (ThermoFisher scientific) was used (1300 V, 10 ms, 4 pulse) with Opti-MEM as a resuspension buffer.

### Genomic DNA isolation of PGCs, sperm and blood samples

For DNA isolation, 5 × 10^5^ PGCs were pelleted and washed twice in PBS (1000 rpm, 4 min). Genomic DNA was extracted using alkaline lysis. The cell pellet was dissolved in 30 µl 100 mM NaOH and heated up to 95 °C for 5 min. Afterwards 100 µl 75 mM Tris–HCl pH 7.5 was added and mixed. After centrifugation (1,000 rpm, 4 min) the DNA in the supernatant was quantified (NanoDrop ND-1000) and stored at − 20 °C.

Sperm samples (50–100 µl) were washed with 70% ethanol until the solution became clear. The pellet was resuspended in 500 µl lysis buffer (50 mM Tris–HCl, pH 8; 100 mM NaCl; 100 mM EDTA; 1% SDS) with 2% proteinase K (10 mg/ml) and 20 mM dithiothreitol (DTT) and incubated overnight at 56 °C. Samples were centrifuged (14,000 rpm, 15 min) and the DNA from the supernatant was precipitated with ethanol. The pellet was suspended in 0.1×  Tris–EDTA.

Blood samples were taken from 7-day-old chicks and DNA from filter paper was isolated as described before^49^.

### PCR sexing and genotyping of PGC-lines

PGCs, chicken embryos and hatched chicks were sexed using primers specific for the W- and Z-associated CHD1 gene (Fig. S4, S5). Primers were used as described with modifications^50^. Primer sequences are listed in Table S5. PCR conditions were an initial denaturation of 95 °C for 2 min, 94 °C for 45 s, 57 °C for 45 s, 72 °C for 45 s and 35 cycles and a final extension of 72 °C for 5 min (Promega GoTaq Polymerase). A 25 µl PCR reaction mixture containing 14 µl ddH_2_O, 5 µl 5× GoTaq reaction buffer, 0.8 µl each forward primer (20 µM), 1.6 µl of reverse primer (20 µM), 1.5 µl MgCl_2,_ 0,5 µl dNTPs (10 mM), 0.13 µl GoTaq Polymerase (Promega) and 0.25 µl TMAC was prepared. 25 ng sample DNA was used.

Primer for genotyping of the blue-layer allele were used as listed (Table S6; Fig. S6). The PCR amplification was performed using 25 ng of genomic DNA and the QIAGEN Multiplex PCR Kit. A 10 µl multiplex PCR reaction mixture containing 5 µl 2× Multiplex PCR Master Mix (Qiagen), 0.75 µl reverse Primer (10 µM), 0.5 µl each forward primer (10 µM), 2 µl ddH_2_O and 2 µl sample DNA was set up. Annealing temperature was 63 °C.

To identify the stable integration of the Venus reporter gene (Table S7) in the chicken genome a Venus-PCR was performed with the Promega GoTaq Polymerase protocol according to the manufacturer's guidelines and with an annealing temperature of 59 °C.

Reaction products were resolved using a 1.5% ultrapure agarose (Invitrogen) gel electrophoresis run at 80 V for 45 min in 1× TBE-buffer, and visualized using a transilluminator.

### Injection of PGCs and embryo culture in surrogate eggshells

In total, 5000 PGCs in a volume of 3 µl manipulations-medium were injected into the dorsal aorta (via the anterior omphalomesenteric vein) of stage 14–16 HH (2.5 d) recipient chicken embryos^47^. After injection the embryo was transferred to a surrogate shell (25–30 g heavier than the donor eggs) and incubated for 21 days until hatching^51, 52^. Injected eggs were incubated in a motor breeder (Brutmaschinen-Janeschitz GmbH (Bruja), Germany) at 37.8 °C ± 2 °C and 40–60% relative humidity with hourly rocking through a 30° angle until day 18.

### Germline transmission potential of cultured and transfected PGCs

The evaluation of PGC colonization and direct Venus fluorescence was analyzed in gonads of 10-day old chicken embryos. For fluorescence microscopy, an Olympus (SZX16) fluorescence stereo microscope equipped with a high-resolution digital camera (Olympus DP74 CMOS), a light source X-Cite 120 Q, and an excitation filter of 460–490 nm, a band pass emission filter of 515–550 nm and a dichroic mirror DM505 (Olympus) were used for detection of Venus fluorescent cells.

The membrane integrity and Venus-fluorescence of sperm (germline chimera) were identified with flow cytometry (Gallios Cytometer 1.2, Beckman Coulter). Ejaculates were collected by dorso-abdominal massage once a week throughout a period of 5 weeks. The ejaculates were directly diluted (1:5–1:10) with HS1 extender to a concentration of 50 × 10^6^ sperm/ml^53^. A staining reaction Master mix with 480 µl HS1 extender and 3 µl To-PRO-3 was prepared for each sample. Per reaction 10–20 µl (5 × 10^5^–1 × 10^6^) of diluted sperm were pipetted into the mixture and incubated for 15 min at 17 °C. In total, 100.000 sperm of each ejaculate were evaluated in double. The average (arithmetic mean) of the five measurements per rooster were calculated (Table 3). As a negative control sperm of a non-treated (Venus-negative) rooster was used.

## Supplementary information

Supplementary Informations.
