# Supplementary material for: Cultivation and characterization of primordial germ cells from blue layer hybrids (Araucana crossbreeds) and generation of germline chimeric chickens
Source: Sci Rep. 2021 Jun 21;11:12923. doi: 10.1038/s41598-021-91490-y (PMC8217269; doi:10.1038/s41598-021-91490-y)
Supplement: Supplementary file 1 — Supplementary Informations. [file 41598_2021_91490_MOESM1_ESM.docx]

**Supplementary information**

**Cultivation and characterization of primordial germ cells from blue layer hybrids (Araucana crossbreeds) and generation of germline chimeric chicken**

Stefanie Altgilbers^1^, Sabine Klein^1^, Claudia Dierks^2^, Steffen Weigend^2^, Wilfried A. Kues^1^

Friedrich-Loeffler-Institut, Institute of Farm Animal Genetics,

Dept. Biotechnology^1^ and Dept. of Genetic Ressources^2^, 31535 Neustadt, Germany

Fig. S1 PGC growth curves

A frozen male and female PGC aliquot from each genotype was thawed. The cell count was initially set to 100.000 cells and the cell count was determined over 4 days using a hemocytometer. The doubling time ranges from 25 to 36 hours (Table S2).


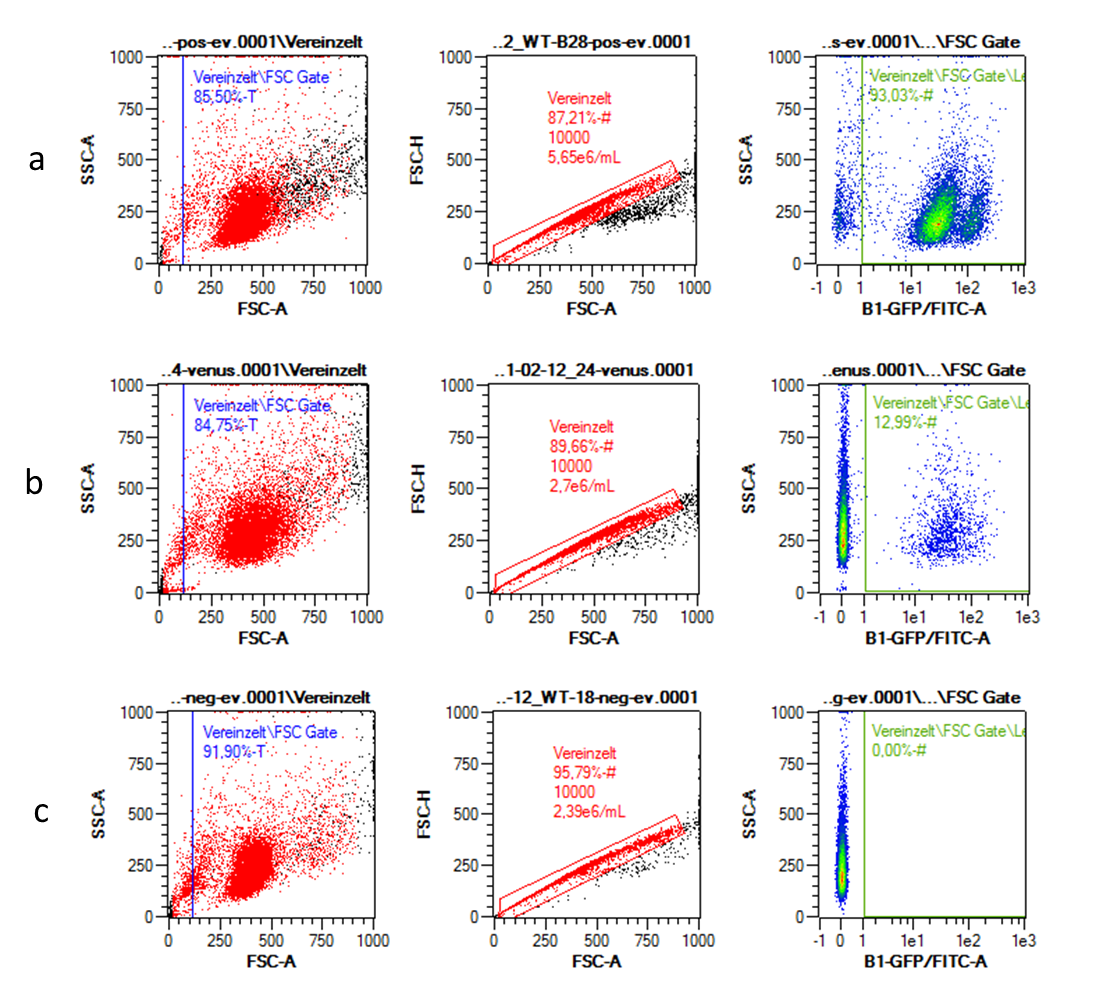


Fig. S2 Venus-fluorescence of PGCs transfected with Venus-transposon in presence and absence of transposase

a) Post-transfection status of FACS sorted PGCs transfected with Venus-transposon and SB-transposase. 93 % of PGCs with stable integrated Venus-gene;

b) post-transfection status of PGCs transfected with Venus-transposon and SB-transposase. 12.9 % of PGCs with stable integrated Venus-gene;

c) post-transfection status of PGCs transfected with Venus-transposon and without SB-transposase. No Venus-fluorescence is detectable.


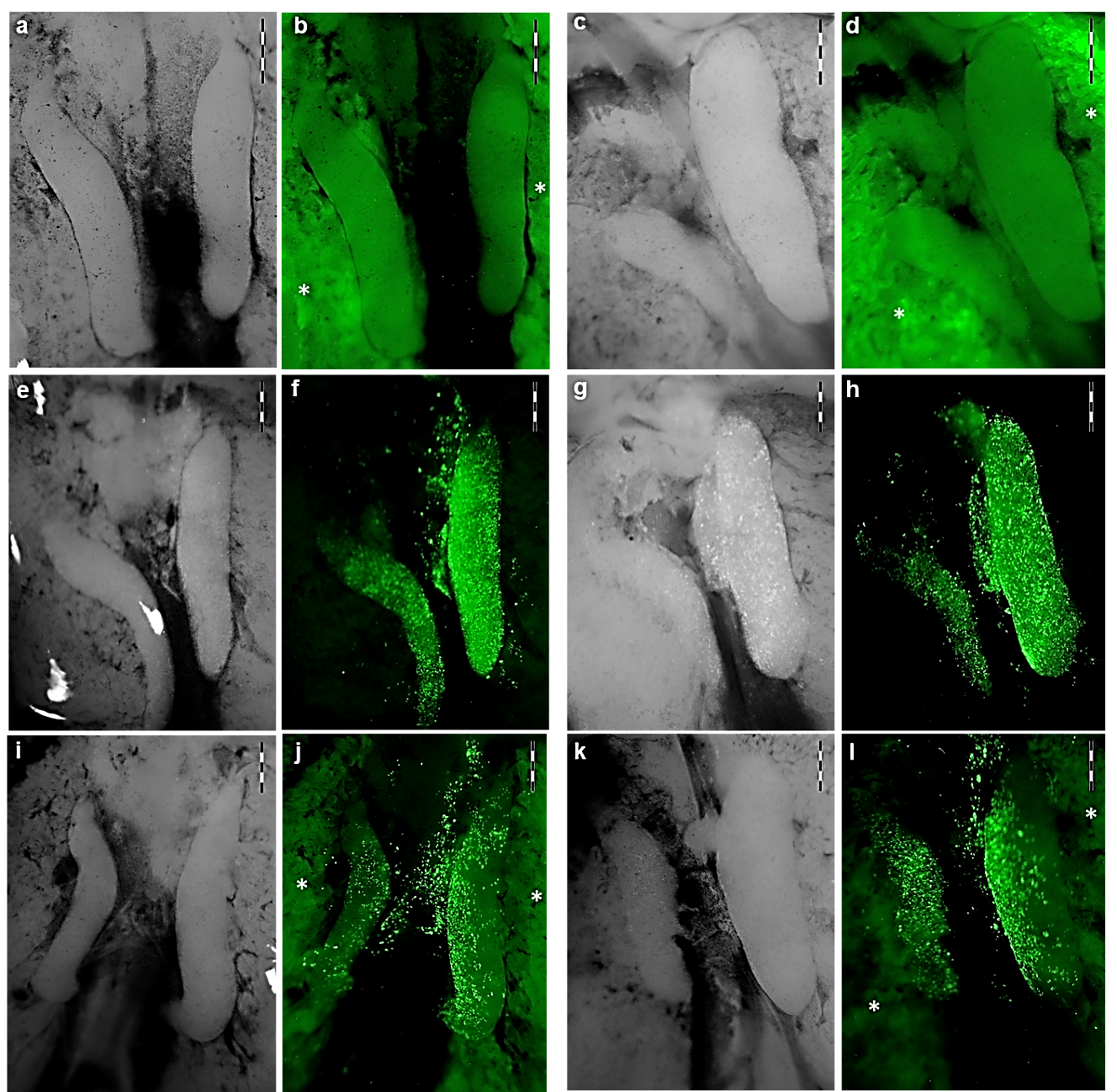


Fig. S3 Venus reporter transgenic PGCs in chicken gonads at day 10 of embryonic development

post-injection status of E10 gonads injected with e-h) homozygous blue-allele bearing PGCs (Venus-positive) i-l) nullizygous blue-allele bearing PGCs (Venus-positive)

a) Microscopic image of male gonads (Brightfield); b) autofluorescence of male gonads and mesonephros (wildtype, Venus-negative);

c) microscopic image of female gonads (Brightfield) d) autofluorescence of male gonads and mesonephros (wildtype, Venus-negative);

e) microscopic image of male gonads (Brightfield); f) green fluorescence of male gonads;

g) microscopic image of female gonads (ovary and rudimentary right ovary); h) green fluorescence of female gonads;

i) microscopic image of male gonads (Brightfield); j) green fluorescence of male gonads;

k) microscopic image of female gonads (ovary and rudimentary right ovary); l) green fluorescence of female gonads.

* autofluorescence of the mesonephros, (a-l: scale bar: 1000 µm)

Fig. S4 Sexing PGCs

1,3 Female PGC lineage;

2,4 male PGC lineage;

5 female control;

6 male control;

7 negative control (blank).


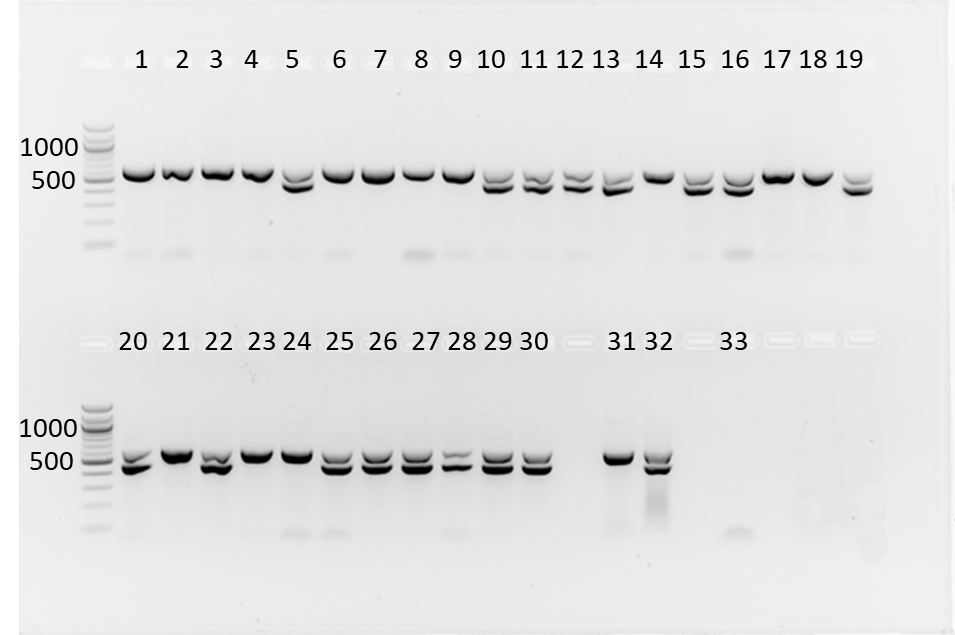


Fig. S5 Sexing 7-day old chickens

1-30 Male and female chicks;

31 male control;

32 female control;

33 negative control (blank).


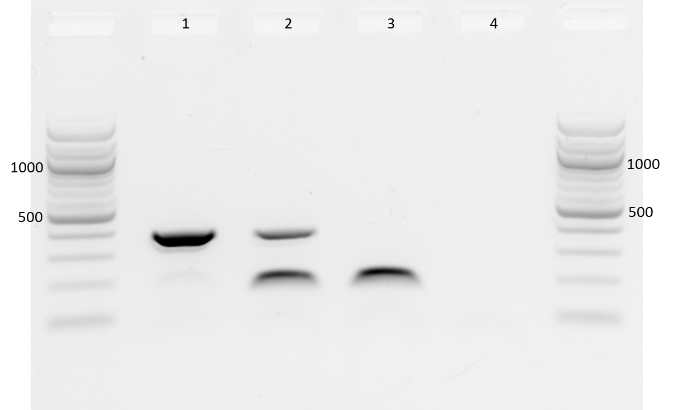


Fig. S6 PCR genotyping (EAV-HP)

1 Nullizygous blue-allele;

2 heterozygous blue-allele;

3 homozygous blue-allele;

4 negative control (blank).

Table S1 Growth performances of the established PGC lineages

| cell line | sex | genotyp (blue allele) | growth performance* |
| --- | --- | --- | --- |
| b10 | male | heterozygous | 40 |
| b14 | male | heterozygous | 37 |
| b23 | male | heterozygous | 50 |
| b48 | male | heterozygous | 49 |
| g14 | male | heterozygous | 36 |
| g20 | male | heterozygous | 45 |
| g23 | male | heterozygous | 62 |
| g24 | male | heterozygous | 46 |
| g31 | male | heterozygous | 62 |
| g34 | male | heterozygous | 43 |
| g41 | male | heterozygous | 62 |
| b15 | female | heterozygous | 50 |
| b45 | female | heterozygous | 38 |
| b50 | female | heterozygous | 66 |
| b51 | female | heterozygous | 66 |
| b54 | female | heterozygous | 66 |
| b6 | female | heterozygous | 49 |
| g18 | female | heterozygous | 47 |
| g2 | female | heterozygous | 47 |
| g27 | female | heterozygous | 60 |
| g32 | female | heterozygous | 43 |
| g4 | female | heterozygous | 48 |
| b1 | male | homozygous | 53 |
| b12 | male | homozygous | 56 |
| b26 | male | homozygous | 50 |
| b28 | male | homozygous | 37 |
| b29 | male | homozygous | 38 |
| b30 | male | homozygous | 39 |
| b41 | male | homozygous | 38 |
| g1 | male | homozygous | 36 |
| g29 | male | homozygous | 60 |
| g35 | male | homozygous | 62 |
| g8 | male | homozygous | 47 |
| b11 | female | homozygous | 51 |
| b52 | female | homozygous | 66 |
| g26 | female | homozygous | 43 |
| b42 | male | nullizygous | 49 |
| b5 | male | nullizygous | 50 |
| g17 | male | nullizygous | 35 |
| g19 | male | nullizygous | 47 |
| g22 | male | nullizygous | 43 |
| g3 | male | nullizygous | 62 |
| g37 | male | nullizygous | 62 |
| g40 | male | nullizygous | 62 |
| g5 | male | nullizygous | 35 |
| g21 | female | nullizygous | 43 |
| g39 | female | nullizygous | 43 |

*days to reach 1,5 x10⁶ cells

b = blood-derived PGC cell lineage, g = gonadal-derived cell lineage

Table S2 Cell counts and doubling time of PGC

|  | PGC line (blue-allele) | | | | | |
| --- | --- | --- | --- | --- | --- | --- |
| Day in culture | nullizygous blue-allele  female (G39) | nullizygous blue-allele  male (G40) | heterozygous  blue-allele  female (G32) | heterozygous  blue-allele  male (G24) | homozygous  blue-allele  male (B29) | homozygous  blue-allele  female (G26) |
| 1 | 100.000 | 100.000 | 1000.000 | 1000.000 | 100.000 | 100.000 |
| 2 | 142.000 | 155.000 | 158.000 | 193.000 | 181.250 | 202.000 |
| 3 | 247.500 | 290.000 | 265.000 | 277.500 | 380.000 | 375.000 |
| 4 | 396.000 | 493.000 | 424.000 | 499.500 | 722.000 | 675.000 |
| Doubling time (h) | 36 | 31 | 35 | 31 | 25 | 26 |

Table S3 Migration efficiency of Venus-positive PGCs

| Cell line | | | No. of embryos injected | No. of embryos  examined at day 10 | No. of Venus-positive gonads (%) | Sex of embryo | |
| --- | --- | --- | --- | --- | --- | --- | --- |
| days in culture | sex | genotype |  |  |  | male | female |
| 65 | male | homozygous blue-allele | 43 | 35 | 35 (100%) | 17 | 18 |
| 90 | male | nullizygous blue-allele | 44 | 42 | 41 (99%) | 26 | 16 |

Table S4 PCR primer sequences for stemness and germline specific transcripts (Whyte et al. 2015)

| Primer | Forward | Reverse | Amplicon (bp) |
| --- | --- | --- | --- |
| GAPDH | 5'- CCTCTCTGGCAAAGTCCAAG -'3 | 5'- CATCTGCCCATTTGATGTTG -'3 | 200 |
| DDX4 | 5'-TCCATCTTTGCATGTTATCAGTCAGG-'3 | 5'-AATCCCGCCCTGCTTGTATAACAG-'3 | 214 |
| POU5 | 5'- GGCTCAATGAGGCAGAGAAC -'3 | 5'- GGACTGGGCTTCACACATTT-'3 | 157 |
| NANOG | 5'- AGCAGACCTCTCCTTGACCA -'3 | 5'- TTCCTTGTCCCACTCTCACC -'3 | 186 |
| DAZL | 5'- TCCCAGAGCCCACACAGATG -'3 | 5'- AAGTGATGCGCCCTCCTCTC -'3 | 160 |

Table S5 Primer sequences for sexing (Fridolfsson and Ellegren 1999)

| Primer | Sex PCR | Amplicon |
| --- | --- | --- |
| F1 | 5'- GTTACTGATTCGTCTACGAGA -'3 | 447 bp (CHD-W)  593 bp (CHD-Z) |
| F2* | 5'- GCTACTGATTCGTCTGCGAGA -'3 |  |
| R | 5'- ATTGAAATGATCCAGTGCTTG -'3 |  |

*Primer F2 was modified by Dierks et. al (unpublished data) according to reference sequences for chicken Z chromosome.

Table S6 Primer sequences for genotyping of the blue egg allele (Wragg et al. 2013)

| Primer | EAV-HP PCR (blue allele or non-blue allele) Wragg | Amplicon (bp) |
| --- | --- | --- |
| F | 5'- GCATTTCACAAACGGGTGTA -'3 | 364 / 167 |
| R1 | 5'- CAAAACCACAAAGGTAATGTTCA -'3 |  |
| R2 | 5'- CCCAGCAGTAAGCCCTACAT-'3 |  |

Table S7 Primer sequences for amplification of the Venus reporter

| Primer | Forward | Reverse | Amplicon (bp) |
| --- | --- | --- | --- |
| Venus | 5'- CTTGTACAGCTCGTCCATGC -'3 | 5'- TCCTCGATGTTGTGCCTGAT -'3 | 216 |

Fridolfsson AK, Ellegren H (1999) A simple and universal method for molecular sexing of non-ratite birds J Avian Biol 30:116-121

Whyte J et al. (2015) FGF, Insulin, and SMAD signaling cooperate for avian primordial germ cell self-renewal Stem Cell Rep 5:1171-1182 doi:10.1016/j.stemcr.2015.10.008

Wragg D et al. (2013) Endogenous retrovirus EAV-HP linked to blue egg phenotype in Mapuche fowl PLoS One 8:e71393 doi:10.1371/journal.pone.0071393
